# Dissecting executive dysfunction in children: The role of psychosocial disabilities through Bayesian and ANOVA approaches

**DOI:** 10.1371/journal.pone.0343809

**Published:** 2026-04-06

**Authors:** Soo Eun Chae

**Affiliations:** Department of Education, Graduate School of Kangwon National University, Gangneung-si, Gangwondo, South Korea; City University of New York, UNITED STATES OF AMERICA

## Abstract

This study investigates the relationship between executive dysfunction and psychosocial disabilities in children aged 8–11, utilizing data from the Korean Child Panel Study collected between 2015 and 2018. The dataset, consisting of 1,196 valid responses, was analyzed using a Latent Growth Model, which revealed that a quadratic model best fit the data. Results indicated that executive dysfunction scores plateaued until age 10 before slightly declining. Children with psychosocial disabilities showed significantly higher levels of executive dysfunction than their peers without such disabilities, with a notable increase over time. We compared the average executive dysfunction levels between children with no psychosocial disorders (N = 1,174) and those with psychosocial disorders (N = 22) using repeated measures ANOVA. To address the small sample size of the psychosocial disorders group, both Bayesian repeated measures analysis and repeated measures ANOVA were performed. The ANOVA identified significant interaction and main effects, while the Bayesian analysis highlighted significant main effects. This discrepancy underscores the need for nuanced methodologies when dealing with small populations. The study confirms a positive correlation between psychosocial disabilities and executive dysfunction observed internationally, emphasizing its relevance to Korean children. These findings underscore the importance of early recognition and intervention for children with psychosocial and cognitive difficulties.

## Introduction

Executive dysfunction refers to impairments in self-regulatory behaviors that aim to optimize goal-oriented actions. Key elements of executive function include inhibition, working memory, and cognitive flexibility [1,2]. The developmental trajectory of executive functions generally shows progressive enhancement during adolescence, followed by a gradual decline in older adulthood, though empirical findings on this progression are not universally consistent [3,4]. The complexity of this domain is particularly evident when examining the neuroanatomical correlates underlying its subcomponents. Impairments in executive functions are notably linked to damage within the frontal lobes [5]. Furthermore, individuals with superior executive functions often have increased volumes of the frontal cortex, with growth continuing into late adolescence before a slow decline begins [6]. Comparative analyses between individuals in early adulthood and those over 60 reveal a diminishing distinction among the subcomponents of executive functions with advancing age [7].

The study of executive dysfunction is particularly important in understanding various psychosocial disorders in children and adolescents. Executive dysfunction in these populations highlights the need for focused research on the developmental patterns of executive functions in relation to psychosocial disorders. The causes of executive dysfunction are intricately linked to conditions such as autism, Attention Deficit Hyperactivity Disorder (ADHD), cognitive impairments, and depression, requiring in-depth exploration [8].

Investigating executive dysfunction, which manifests in a range of psychological and social disorders, especially among youth, necessitates a detailed examination of the developmental trajectories of executive functions within these contexts. This investigation is crucial for early diagnosis and the development of proactive intervention strategies to support the growth and development of affected children and adolescents. Key conditions like autism, ADHD, cognitive impairments, and depression are strongly associated with discrepancies in executive functions [9,10]. To better understand these associations, a comprehensive analysis of seminal research contributions in this field was undertaken.

### Analysis of changes in executive dysfunction by subdomain

Researchers have largely recognized executive function or dysfunction as comprising a dual or multiple structure [11–15]. The complexity of executive function structure implies limitations in analyzing developmental or changing trends simply by aggregating executive function scores. Therefore, it is necessary to analyze the subdomains of executive function.

While scholars may use slightly different operational definitions of executive function, it generally includes areas such as attention, working memory, and inhibition control (or self-regulation) [2]. A notable study on changes in executive function subdomains by age [14] found that these changes and patterns varied across different subdomains according to age. This study analyzed the performance of 688 children and adolescents aged 6–15 years using a cross-sectional design. The results showed that older students generally performed better on tasks such as memory task accuracy, inhibition cost, and switch cost [14]. Inhibition cost refers to how quickly responses to contradictory situations are compared to automatic responses, indicating cognitive performance, control, and speed related to inhibition. For instance, the inhibition cost for second-year kindergarten (K2) students was generally higher than that for older students [14]. This implies that younger students take longer and have slower responses in dealing with contradictory situations compared to automated situations. Switch cost indicates the delay in response time for tasks requiring a transition compared to tasks not requiring a transition. It was found that younger students had higher switch costs, indicating higher delays, especially in tasks requiring a transition.

Executive dysfunction, on the other hand, refers to difficulties in these same areas of executive function, often seen in psychological conditions such as ADHD and Autism Spectrum Disorder (ASD). Research integrating behavioral and neuroscientific perspectives has illuminated the connections between these conditions and executive dysfunction [2]. Despite these advancements, there is a noticeable lack of studies that specifically explore how patterns of executive dysfunction evolve over time, particularly in relation to the presence or absence of these disorders, and especially where longitudinal data is involved. This gap highlights the importance of further research in this area to better understand the changes in executive dysfunction over time and in the context of psychological disorders.

By comparing the operational definitions of executive function and executive dysfunction, we can see that while executive function involves the effective performance of cognitive tasks, executive dysfunction pertains to the challenges and impairments in performing these tasks. Understanding both concepts and their development over time is crucial for devising better interventions and support mechanisms for individuals with executive dysfunction, particularly those with ADHD and ASD.

### Bayesian analysis vs. Analysis of Variance (ANOVA)

To explore the relationship between psychosocial disabilities in childhood and changes in executive dysfunction, this study employs both Bayesian analysis and Analysis of Variance (ANOVA). Bayesian analysis provides a probabilistic framework that incorporates prior information and updates the probability as new data becomes available, making it particularly useful for small sample sizes or complex models with many parameters. ANOVA, on the other hand, is a traditional statistical method that partitions variance into components attributable to different sources, allowing for the comparison of means across groups. Comparing these two methods offers insights into the robustness of the findings and the potential advantages or limitations inherent in each approach. This dual-method approach ensures a comprehensive analysis and validation of results.

### Research questions

Research Question 1: What are the patterns and magnitudes of changes in executive dysfunction scores during childhood (ages 8–13)?

Research Question 2: Do changes in executive dysfunction scores during childhood differ based on the presence of psychological disorders?

## Methods

### Data collection

This study utilized data from the Panel Study on Korean Children (PSKC), an ongoing longitudinal project conducted by the Korea Institute of Child Care and Education since 2008. The survey targets newborns born in medical institutions nationwide between April and July 2008, with annual follow-up surveys conducted until 2027. The survey covers various aspects including child characteristics, parental characteristics, family characteristics, school characteristics, characteristics of childcare support services, community characteristics, and characteristics of childcare support policies. In this study, we focused on child characteristics, specifically executive dysfunction and diagnosis of psychological disorders.

Executive dysfunction was assessed using the “Brief Self-Report of Executive Functioning in Children and Adolescents” questionnaire developed by Song Hyun-joo [16]. This questionnaire consists of a total of 40 items assessing difficulties in planning-organization, behavioral control, emotional control, and inattention, with responses rated on a 3-point scale. The self-report format was chosen to capture the children’s own perspectives on their executive functioning, which is crucial for understanding their subjective experiences and self-awareness of their difficulties. Further commonality analyses indicated that behavioral ratings of executive function accounted for more unique variance in predicting cognitive disorders and also displayed overlap with the performance based measures [17]. The data used in this study were obtained from parental responses from the 8th to the 11th year of the survey. To ensure item reliability through respondent consistency, data directly obtained from children after the 13th year were not utilized. The internal consistency reliability (McDonald’s ω) for the data used in this study, categorized by the scale name and subdomains (planning-organization difficulty, behavioral control difficulty, emotional control difficulty, attention difficulty), was satisfactory (see Table 1).

**Table 1:** Items and internal consistency (McDonald’s ω) for subgroups with executive function difficulties included in the Korean Children’s Panel.

Category	Program Content	8th (2015)	9th (2016)	10th (2017)	11th (2018)
**Self-Control Group**	1. I can control my mind and maintain calm.	0.875	0.885	0.881	0.891
	11. When I am upset, I know how to calm myself down without getting agitated.				
**Aggression-Control Group**	12. I understand that expressing my anger by throwing and breaking things does not solve any problems.	0.857	0.849	0.838	0.854
	22. When I am angry, I try not to hurt others with my words or actions.				
**Social Skills Group**	23. I try to understand and sympathize with friends.	0.893	0.904	0.909	0.899
	30. I try to make amends after a fight.				
**Emotion-Regulation Group**	31. I try to express my feelings in words rather than keeping them inside.	0.897	0.900	0.914	0.911
	40. When I am sad, I can express it in ways other than crying.				

For the results analysis, the sum of scores for 40 items pertaining to executive dysfunction was utilized. The original data included a total of 2,152 cases, out of which 1,196 cases (56%) that had at least one non-response to the executive dysfunction scale from the 8th to the 11th survey were excluded due to incomplete responses. A demographic difference was investigated between the data that responded to all items on executive dysfunction and the data that showed incomplete responses. It was found that the incomplete respondents had a very low occurrence of valid values across all three variables: gender, father’s highest educational attainment, and subjective socioeconomic status (valid values were either 4 or 0). In other words, incomplete respondents consistently showed non-response across most other variables. Consequently, the data from incomplete respondents were deleted. The analysis showed no statistically significant differences in executive dysfunction responses and non-responses when categorized by gender, father’s highest education level, and subjective socioeconomic status: gender *χ*^*2*^ = 0.350, *p* = 0.554, father’s highest education *χ*^*2*^ = 0.371, *p* = 0.985, socioeconomic status *t* = 0.624, *p* = 0.533. Thus, it was speculated that the deletion of non-responders would not significantly distort the data (Table 2).

**Table 2:** Descriptive statistics of incomplete and complete respondents with executive dysfunction (based on 2020 responses).

	Gender		Father		Adolescents’ subjective socioeconomic status	
	Responses	Non-responses	Responses	Non-responses	Responses	Non-responses
**Number of Observations**	1393	4	1348	4	1359	0
**Executive Dysfunction Group**	290	465	335	465	324	469
**Mean**	1.493	1.500	5.390	5.750	6.611	NaN
**Standard Deviation**	0.500	0.577	1.004	1.258	1.528	NaN

Psychosocial disorder diagnosis statistics were based on the latest information from responses in 2020. Based on the responses from 2020, the number of sincere respondents who reported having been diagnosed at least once between 2011 and 2020 was a total of 22 cases. The types of psychosocial disorder diagnoses included a total of 10 categories (1. Communication, 2. Autism, 3. ADHD, 4. Specific Learning, 5. Motor, 6. ODD, 7. Elimination, 8. Feeding Eating, 9. Anxiety, 10. Other).

The research protocol, including the ethical and scientific validity of the data analysis, the process of obtaining consent from participants, the safety of the research subjects, and measures for the protection of personal information, was reviewed and approved by the Institutional Review Board (IRB) of the Korean Children’s Panel Study Institution. Final IRB approval was granted on June 16, 2015 (Approval No. KICCEEIRB-2015-03, KICCEEIRB-2016-07, KICCEEIRB-2017-05, KICCEEIRB-2018-02). Additionally, the research received an exemption from review by the author’s affiliated institution (GWNUIRB-R2024-23) due to its use of secondary data, which involves minimal risk. Given the nature of the primary research subjects as minors, who are considered ‘vulnerable research participants’, our study was conducted in strict adherence to guidelines aimed at protecting the autonomy of the children and ensuring their voluntary consent. All necessary steps were taken to provide sufficient information to the participants prior to their involvement in the study to facilitate an informed decision about their participation.

### Analysis

**Fig 1:**
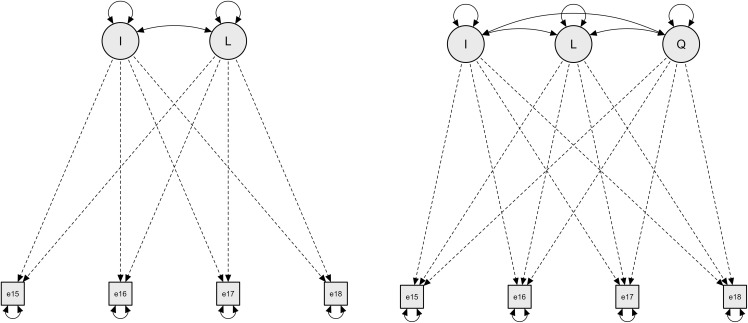
First- and second-order latent growth models of executive dysfunction (ages 8–11).

In this study, we analyzed the trend of change in executive dysfunction across all 1,196 cases (latent growth model) and examined the impact of the presence or absence of psychosocial disorders on development. We also compared the average level of executive dysfunction between the group with no psychosocial disorders (*N* = 1,174) and the group with psychosocial disorders (*N* = 22) using a repeated measures analysis of variance. After controlling for the presence of psychosocial disorders (group without psychosocial disorders vs. group with psychosocial disorders) as a predictive variable, we tested both a linear model and a quadratic model to understand how executive dysfunction changes over time (see Fig 1). The benefit of testing a linear model is that it allows us to observe a consistent, straightforward relationship between age and executive dysfunction, highlighting a constant rate of change. In contrast, a quadratic model helps capture more complex, non-linear relationships, allowing us to see if the rate of change accelerates or decelerates at different ages. By testing both models, we can better capture the nuances in how executive dysfunction evolves, providing a more comprehensive understanding of its progression. This dual approach enhances our ability to identify critical periods for intervention and support, particularly for individuals with ADHD, ASD, and other related conditions. The best model was selected according to the adequacy criteria for each model’s data-model fit index (RMSEA < .08; CFI > .90; TLI > .90; SRMR < .08) and the parsimonious rule.

A repeated measures ANOVA (Analysis of Variance) was conducted to compare the average levels of executive dysfunction between groups without psychosocial disorders (*N* = 1,174) and the psychosocial disorder response group (*N* = 22). According to the G power analysis, the minimum required number of cases to maintain a power (1-*β*) of 0.80 for repeated measures ANOVA was 22 per group. To compensate for the issues related to the small cell size (*N* = 22), both Bayesian repeated measures analysis and repeated measures ANOVA were performed. For both the latent growth model analysis and repeated measures analysis, JASP 0.17.1 (JASP Team, 2023) was used. The composition of the disorder response group by type is presented in Table 3.

**Table 3:** Composition of respondents by type of psychosocial disorder.

	Disorder type	2011	2012	2013	2014	2015	2016	2017	2018	2019	2020	Total
**1**	**Communication**	1	1	2			1	1				6
**2**	**Autism Spectrum**				1							1
**3**	**ADHD**				1(1)	1(1)		1				3(2)
**4**	**Specific Learning**											0
**5**	**Motor**		1			1(1)		1(1)	1	1		5(2)
**6**	**ODD**								(1)			(1)
**7**	**Elimination**		1									1
**8**	**Eating**											0
**9**	**Anxiety**						(1)		(1)			(2)
**10**	**Other**								2	1		3

The number in parentheses is the number of cases diagnosed with multiple psychosocial disorders.

## Results

### Developmental trend of executive dysfunction

**Fig 2:**
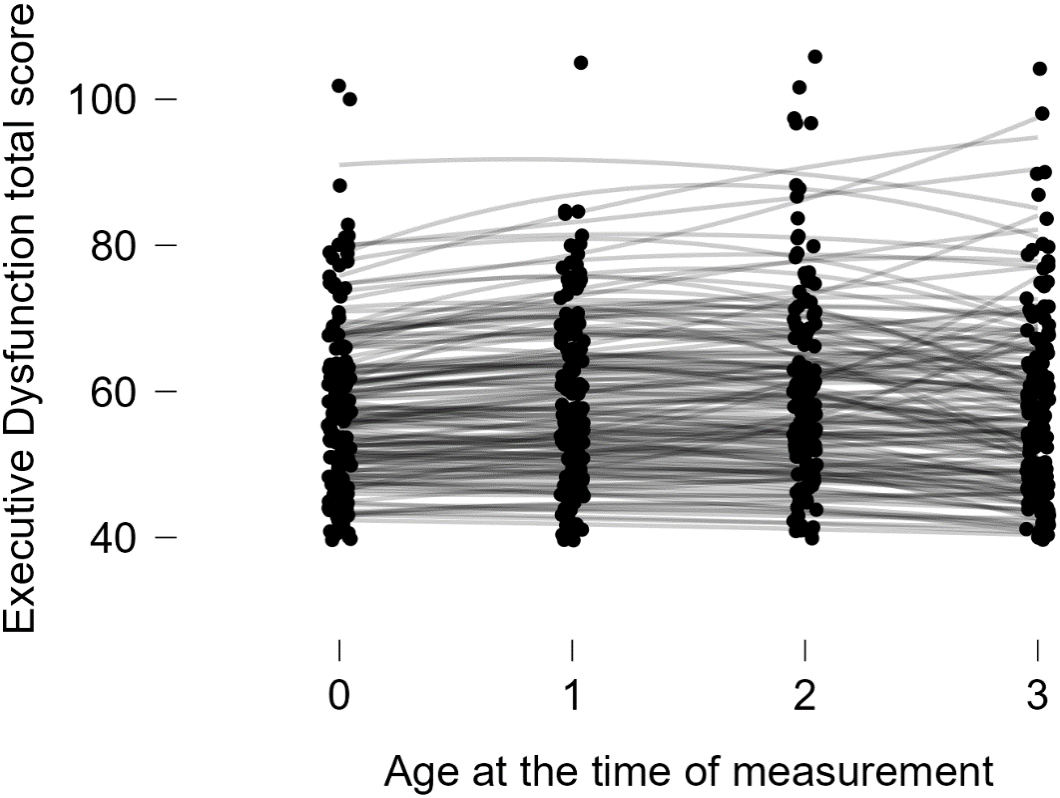
Total score for executive dysfunction by age at each measurement wave, from T0 (aged 8, 2015) through T3 (aged 11, 2018).

The results of the Latent Growth Model analysis, with the presence of psychosocial disorders as a predictive variable, are as follows. Among the Linear Model and the Quadratic Model, the Quadratic Model was deemed appropriate as it met all the adequacy criteria (RMSEA < .05, CFI > .95, TLI > .95, SRMR < .08, *χ²/df* < 3) (refer to Table 4). In this model, the growth trajectory is considered to form a curve (see Fig 2). As shown in the figure, the scores for executive dysfunction among the subjects remained at a similar level until the age of 10, after which they showed a tendency to gradually decrease.

**Table 4:** Model Fit by latent growth model.

	RMSEA	CFI	TLI	SRMR	χ^2^	df	χ^2^/df
1st *	.103	.973	.961	.040	95.177	7	13.597
**2nd**	.045	.998	.992	.009	6.847	2	3.424

### Bayesian analysis of executive dysfunction by psychosocial disorder status

Because there were only 22 cases in the group diagnosed with psychosocial disorders, traditional growth models encountered issues with singular matrices, especially when analyzing sub-components of executive dysfunctions. Therefore, a Bayesian repeated measures analysis was conducted to compare growth patterns between children with and without psychosocial disorders.

**Fig 3:**
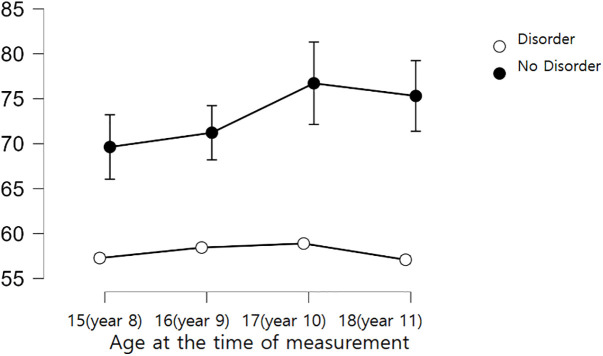
Trends in average executive dysfunction scores based on the presence of psychosocial disorders.

The results of this Bayesian repeated measures analysis, shown in Table 5, indicate distinct trends. Children without psychosocial disorders maintained an average executive dysfunction score of 55.5 up to age 11. In contrast, children with psychosocial disorders showed a gradual increase in executive dysfunction scores, reaching an average of 71 points by age 11 (refer to Fig 3).

**Table 5:** Model of executive dysfunction and psychosocial disorder status, effect comparison, and post-hoc summary.

Models	P(M)	P(M|data)	BFM		BF10	error %
ED + Disorder + ED * Disorder	0.200	0.806	16.650		1.000	
ED + Disorder	0.200	0.194	0.961		0.240	3.895
ED	0.200	8.326 × 10^−9^	3.330 × 10^−8^		1.033 × 10^−8^	2.127
Disorder	0.200	1.020 × 10^−10^	4.081 × 10^−10^		1.265 × 10^−10^	2.673
Null hypothesis model (including study subjects and random slopes)	0.200	4.295 × 10^−18^	1.718 × 10^−17^		5.327 × 10^−18^	1.993
**Effects analysis**						
**Effects**	**P(incl)**	**P(excl)**	**P(incl|data)**		**P(excl|data)**	**BFincl**
Executive Dysfunction	0.600	0.400	1.000		1.020 × 10^−10^	6.534 × 10^+9^
Disorder	0.600	0.400	1.000		8.326 × 10^−9^	8.007 × 10^+7^
Executive Dysfunction * Disorder	0.200	0.800	0.806		0.194	16.650
**Post-hoc summary of mean by model**						
**Variable**	**Level**		**Mean**	**SD**	**95% Credible Interval**	
					**Lower**	**Upper**
Intercept			65.235	1.209	62.779	67.501
Executive Dysfunction	15 (year 8)		−1.543	0.679	−2.894	−0.453
	16 (year 9)		−0.319	0.645	−1.624	0.693
	17 (year 10)		1.714	0.633	0.695	3.002
	18 (year 11)		0.148	0.693	−1.059	1.461
Disorder	0		−7.286	1.210	−9.696	−4.852
	1		7.286	1.210	4.787	9.606
Executive Dysfunction * Disorder	15 (year 8) & 0		1.095	0.591	−0.060	2.305
	15 (year 8) & 1		−1.095	0.591	−2.315	0.049
	16 (year 9) & 0		1.018	0.569	−0.102	2.166
	16 (year 9) & 1		−1.018	0.569	−2.177	0.092
	17 (year 10) & 0		−0.915	0.581	−2.107	0.209
	17 (year 10) & 1		0.915	0.581	−0.220	2.095
	18 (year 11) & 0		−1.198	0.580	−2.356	−0.069
	18 (year 11) & 1		1.198	0.580	0.058	2.345

**Note.** ED = Executive Dysfunction; Disorder = Psychosocial Disorder. • *P(M)*: Prior probability of the model, assuming a uniform distribution (0.20) across all tested models. • *P(M|data)*: Posterior probability of the model given the observed data. • *BF*_*M*_: Bayes factor for the model, indicating the relative evidence for a specific model. • *BF*₁₀: Bayes factor representing the evidence for the target model relative to the reference (null) model. • **error** %: The numerical error in the estimation of the Bayes factor. • *P(incl) & P(excl)*: Prior probabilities of including or excluding an effect in the model, respectively. • *P(incl|data) & P(excl|data)*: Posterior probabilities of inclusion or exclusion after observing the data. • BF_*incl*_: Bayes factor for inclusion, quantifying the change from prior to posterior inclusion odds; higher values indicate stronger evidence for including the effect.

To understand the impact of executive dysfunction and psychosocial disorders, various models were compared (see Table 5). The model that included the interaction between executive dysfunction and the presence of psychosocial disorders had strong support from the data, with a Bayesian Factor (BFM) of 16.650, indicating it explains the data significantly better than the null model. A Bayesian factor greater than 10 indicates strong evidence for the model.

The updated inclusion probability (P(inc|data)) for the interaction term was quite high (P = .806). This means there is substantial evidence for an interaction effect. The main effects of executive dysfunction and the presence of psychosocial disorders also had high inclusion probabilities (P = 1) and Bayesian factors (BF_incl_ > 10), indicating strong support for these effects.

The model average post-hoc summary includes estimated values for each variable level. The initial value (intercept) had a posterior mean of 65.235, with a 95% confidence interval of 62.779 to 67.501. The interaction effect of executive dysfunction scores at age 11 and the presence of psychosocial disorders had a posterior mean of 1.198, with a confidence interval of.058 to 2.345, indicating a significant interaction effect.

**Fig 4:**
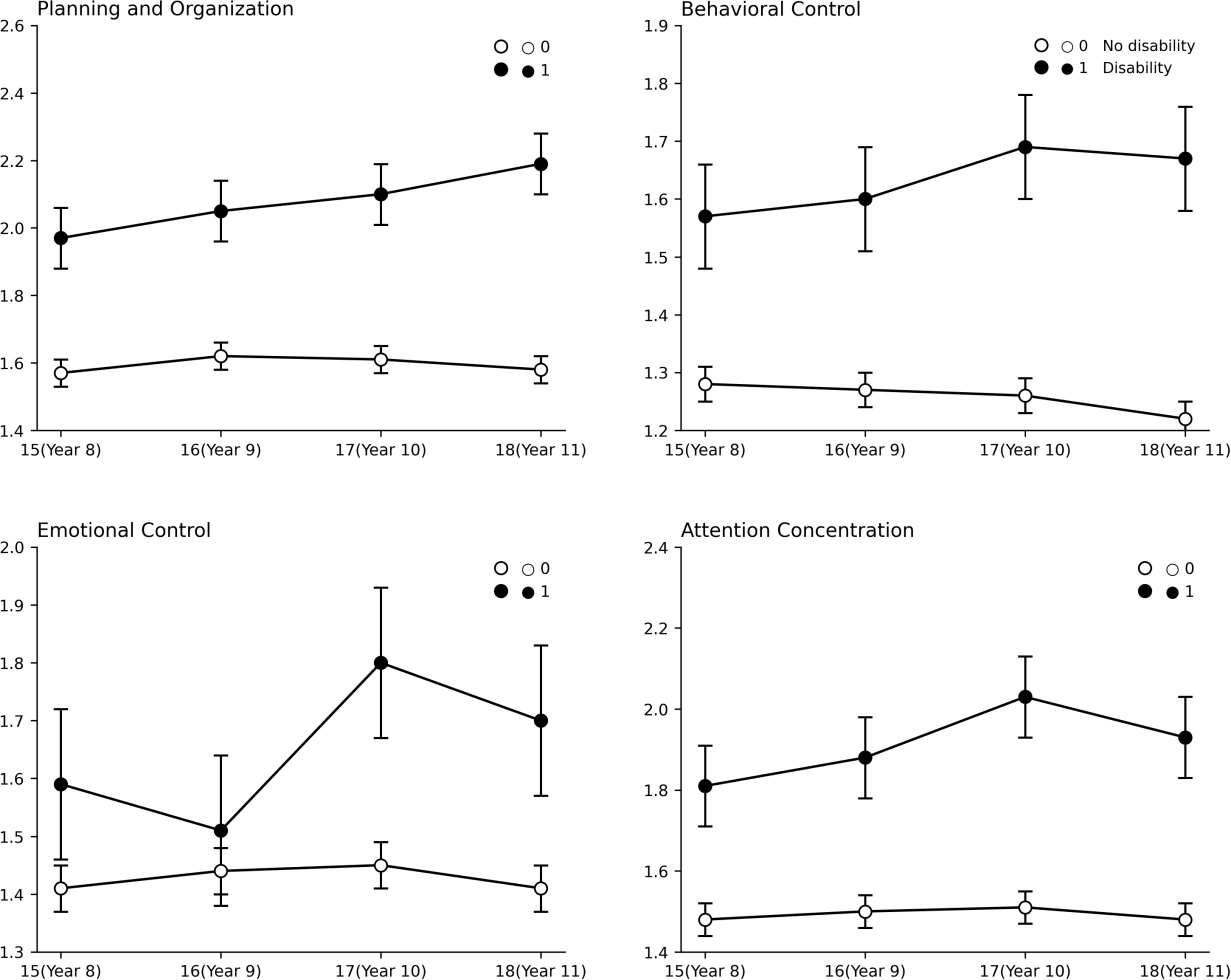
Change in average scores for subcomponents of executive dysfunction depending on psychosocial disorders.

Fig 4 illustrates the trends in average scores for subcomponents of executive dysfunction based on the presence of psychosocial disorders. For children without disorders, average scores for planning-organization difficulties, behavioral control difficulties, and attention concentration difficulties decreased by age 11. Behavioral control difficulties showed a significant and consistent decrease. In contrast, emotional control difficulties in children with psychosocial disorders increased until age 11, with behavioral control and attention concentration difficulties peaking around age 10 before slightly decreasing at age 11. This suggests that while children with psychosocial disorders experience severe difficulties around age 10, some improvement occurs by age 11.

Overall, children without psychosocial disorders showed relatively stable patterns, whereas children with psychosocial disorders exhibited more significant changes. These patterns varied by difficulty area (refer to Table 6). For example, the interaction effect between planning-organization difficulties and the presence of psychosocial disorders had a low inclusion probability (P = .356) and a Bayesian factor (2.215), indicating weak support. However, behavioral control difficulties had a high inclusion probability (P = .920) and a strong Bayesian factor (46.267), indicating significant support for the interaction effect. Emotional control and attention concentration difficulties had low inclusion probabilities (P = .200) and Bayesian factors below the threshold (≤10), indicating weak support for these interactions.

**Table 6:** Results of the Bayesian repeated measures analysis for the executive dysfunction model.

Planning and Organization Difficulties					
Effects	P(incl)	P(excl)	P(incl|data)	P(excl|data)	BFincl
Year of Measurement for ED (Age at Time of Measurement)	0.600	0.400	1.000	2.442 × 10^−15^	2.729 × 10^+14^
Psychosocial Disorder	0.600	0.400	1.000	2.037 × 10^−8^	3.272 × 10^+7^
Year of Measurement for ED (Age at Time of Measurement) ✻ Psychosocial Disorder	0.200	0.800	0.356	0.644	2.215
**Behavioral Control Difficulties**					
**Effects**	**P(incl)**	**P(excl)**	**P(incl|data)**	**P(excl|data)**	**BFincl**
Year of Measurement for ED (Age at Time of Measurement)	0.600	0.400	1.000	3.886 × 10^−14^	1.716 × 10^+13^
Psychosocial Disorder	0.600	0.400	1.000	7.342 × 10^−10^	9.080 × 10^+8^
Year of Measurement for ED (Age at Time of Measurement) ✻ Psychosocial Disorder	0.200	0.800	0.920	0.080	46.267
**Emotional Control Difficulties**					
**Effects**	**P(incl)**	**P(excl)**	**P(incl|data)**	**P(excl|data)**	**BFincl**
Year of Measurement for ED (Age at Time of Measurement)	0.600	0.400	0.865	0.135	4.273
Psychosocial Disorder	0.600	0.400	0.967	0.033	19.693
Year of Measurement for ED (Age at Time of Measurement) ✻ Psychosocial Disorder	0.200	0.800	0.637	0.363	7.024
**Attention Concentration Difficulties**					
**Effects**	**P(incl)**	**P(excl)**	**P(incl|data)**	**P(excl|data)**	**BFincl**
Year of Measurement for ED (Age at Time of Measurement)	0.600	0.400	1.000	3.364 × 10^−6^	198150.933
Psychosocial Disorder	0.600	0.400	1.000	6.554 × 10^−5^	10170.834
Year of Measurement for ED (Age at Time of Measurement) ✻ Psychosocial Disorder	0.200	0.800	0.076	0.924	0.330

ED = Executive Dysfunction; Disorder = Psychosocial Disorder.

In summary, the Bayesian analysis showed strong evidence supporting the inclusion of the interaction term for executive dysfunction. This means that for students with psychosocial disorders, scores for behavioral control difficulties significantly increased, whereas for students without such disorders, these scores gradually decreased.

Although the probability of including the interaction term in the model wasn’t very high, the main effects had strong support. For example, the probability of including the main effects (P = 1) and the Bayesian factor (BFincl > 10) were high for all subcomponents of executive dysfunction. This indicates that the year of measurement and the presence of a disorder have a significant impact on different types of executive dysfunction.

However, emotional control difficulties did not show a high Bayesian factor (BF_incl_ = 4.273, which is less than 10), making it hard to conclude that these difficulties change significantly over the years. This is shown in Fig 4, where the average scores for emotional control difficulties fluctuate, showing no consistent pattern of increase or decrease.

To strengthen the analysis of the limited number of responses related to psychosocial disorders (*N* = 22), we initially conducted a Bayesian repeated measures analysis. According to G power analysis, the required sample size per group to achieve a statistical power of.80 (1-β) for repeated measures ANOVA was determined to be 22 participants. This was necessary to ensure sufficient statistical power for the study.

### Conventional repeated measures ANOVA analysis

The goal of this section is to compare the results from the Bayesian analysis with those obtained from the conventional repeated measures ANOVA.

When examining the interaction effect between disability status and the timing of measurements (Table 7), it was clear that the assumption of sphericity (which is crucial for analyzing within-group effects) was violated. To address this, we applied the Greenhouse-Geisser correction, which adjusts the degrees of freedom. This correction revealed a significant effect of time (*F* = 6.417, *p* < .001) and an interaction effect between time and disability status (*F* = 4.611, *p* = .004). This means that there were notable changes over time in the severity of executive dysfunctions and significant differences in these changes depending on disability status. Specifically, the severity of executive dysfunctions varied significantly over time in children with psychosocial disorders compared to those without, as indicated by a strong interaction effect (*F* = 41.215, *p* < .001).

**Table 7:** Executive dysfunction scores by disability status and measurement time and their interaction effects.

Within Subjects Effects						
Cases	Sphericity Correction	Sum of Squares	*df*	Mean Square	*F*	*p*
ED(Executive Dysfunction)	None	900.849ᵃ	3.000ᵃ	300.283ᵃ	6.417ᵃ	< .001ᵃ
	Greenhouse-Geisser	900.849	2.864	314.596	6.417	< .001
ED * Disorder	None	647.260ᵃ	3.000ᵃ	215.753ᵃ	4.611ᵃ	0.003ᵃ
	Greenhouse-Geisser	647.260	2.864	226.037	4.611	0.004
Residuals	None	167623.358	3582.000	46.796		
	Greenhouse-Geisser	167623.358	3419.034	49.027		
**Between Subjects Effects**						
**Cases**		**Sum of Squares**	***df***	**Mean Square**	***F***	***p***
Disorder		20203.812	1	20203.812	41.125	< .001
Residuals		586589.108	1194	491.281		

Type III Sum of Squares.

ᵃ Mauchly’s test of sphericity indicates that the assumption of sphericity is violated (*p* < .05).

ED = Executive Dysfunction; Disorder = Psychosocial Disorder.

These results are contrasted with the Bayesian repeated measures analysis, which did not show a sufficient probability for including interaction terms, though the main effects were adequately supported.

## Discussion and conclusion

The objective was to explore how the developmental patterns of executive dysfunctions vary depending on the presence of psychosocial disorders. Analysis was conducted on 1,196 cases (non-psychosocial disorder group, *N* = 1,174; psychosocial disorder group, *N* = 22) after excluding non-response data from the 8th (measured in 2015) to the 13th (measured in 2020) wave of the Korean Child Panel study. Although the psychosocial disorder group consisted of a relatively small sample (N = 22) compared to the control group (N = 1,174), we employed Bayesian repeated measures analysis to compensate for the reduced statistical power and to ensure the robustness of our findings in this minority population. The results led to the following conclusions:

First, as adolescents aged, the level of executive dysfunctions initially remained stable before showing a gradual decrease. The overall growth pattern resembled a quadratic curve converging towards the median. In other words, the level of executive dysfunctions tended to decrease with age, showing a gradual curve. These results did not fully support previous studies (Salat et al., 2004) that suggested executive functions improve until adolescence and then decline in adulthood.

As indicated in prior research, executive dysfunctions possess multidimensional properties, necessitating more detailed analysis. This study analyzed developmental patterns for four underlying constructs: planning-organization, behavioral control, emotional control, and attention concentration. While there were some differences between subcomponents, overall, the level of executive dysfunctions decreased as adolescence approached. This result aligns with Lee, Bull, & Ho [14], showing that younger students have relatively lower executive functioning capabilities across all subcomponents.

This study included inhibitory control and attention concentration as subcomponents instead of short-term memory and was based on participants’ self-reports, differing from Lee, Bull, & Ho’s approach [14]. Consequently, a trend of decreasing executive dysfunctions was observed up to age 11, but it was challenging to confirm this trend up to age 13 due to a change in the data collection method. After age 11, the executive dysfunction items in the Korean Child Panel changed to self-report form, leading to difficulties in ensuring data consistency, thus excluding data after age 13 from this study. Consistent measurement tools are needed to support prior research that suggests executive functions, including concentration and inhibitory control performance, improve into mid-adolescence.

Separate from this study, analysis of self-response data from adolescents after age 13 revealed differences in executive dysfunction scores depending on the presence of psychosocial disorders, indicating that adolescents experiencing psychosocial disorders perceived their executive dysfunction to be higher than what their mothers reported. More systematic data structuring and organization are required to encompass the developmental patterns of executive function from childhood to adolescence, as shown in Salat et al. [7] and based on frontal lobe changes in Duncan et al. [5]. That is, further accumulation of data and research findings is necessary to explain the patterns of change in adolescent executive dysfunction.

Secondly, children with psychosocial disabilities exhibited higher levels of executive dysfunction compared to typical children. This correlation has been substantiated by several previous studies [18,19]. Specifically, ADHD (Attention-Deficit/Hyperactivity Disorder) and Autism Spectrum Disorder (ASD) are frequently associated with executive dysfunction [9,10]. Our study confirmed that children with psychosocial disabilities have, on average, higher levels of executive dysfunction than those without such disabilities. ADHD and ASD, as neurodevelopmental disorders, particularly impact frontal lobe functions, which are crucial for executive processes [20,21]. Research has shown that these disorders often involve difficulties in rational thinking and self-regulation, key components of executive functioning [22,23]. Despite extensive research supporting this association, the causal relationship between psychosocial disorders and executive dysfunction has not been definitively established [24,25]. However, our study aligns with international findings, indicating that children with psychosocial disorders in Korea also experience significant executive dysfunction. This consistency across different populations underscores the pervasive impact of psychosocial disabilities on executive functioning.

Thirdly, the level of executive dysfunctions in children with psychosocial disabilities increased more sharply than in typical children. The Bayesian repeated measures analysis of variance showed a statistically significant interaction effect between the presence of psychosocial disorders and changes in executive dysfunctions. Particularly, the change in behavioral control difficulty was clearly different depending on the presence of psychosocial disorders. Specifically, children with psychosocial disorders showed a marked increase in executive dysfunctions around age 11. Neurobiological changes and environmental factors interacting during development could have influenced executive dysfunctions, as Moffitt et al. [26] suggested that imbalances in executive functions development appear early in adolescence, and psychosocial stress can negatively affect executive functions. Moreover, children with psychosocial disabilities generally experience higher stress at school or home, and this accumulated stress is prominent around age 11 in Korea, potentially exacerbating executive dysfunctions [27]. The increase in executive dysfunctions around age 11 can be interpreted as the result of various challenges and stress responses experienced by children with psychosocial disabilities. Moreover, the interaction effect between behavioral control difficulty and age was more distinct compared to other underlying constructs, highlighting that behavioral control problems are significant in addressing psychosocial disabilities.

### Limitations

Although this study incorporated demographic factors (e.g., gender, paternal education, subjective socioeconomic status) in an effort to maintain data consistency, these environmental factors will continue to exist as potential confounding variables of both psychosocial and executive outcomes. More rigorous controls for contextual variables of this nature are therefore warranted to delineate their effects in future research. Additionally, this study focused on a specific developmental window in late childhood (8–11 years) lasting three years. Though these findings are important, they are limited in their ability to describe enduring executive functioning trajectories as children reach late adolescence or adulthood. And finally, although our findings agree with international research that links psychosocial disabilities with executive dysfunction, the findings might differ in their effects, which may be uniquely influenced by the academic and social stressors inherent to the Korean educational environment. That is, the generalizability of these findings to other cultural or institutional contexts needs to be cautiously qualified.

### Policy recommendations and implications

The analysis of developmental patterns of executive dysfunctions based on the presence of psychosocial disabilities can significantly impact mental health policies and child welfare programs. The findings indicate that children with psychosocial disabilities exhibit a higher average level of executive dysfunctions compared to those without, with an increasing trend up to age 11. This contrasts with children without disabilities, whose executive dysfunction levels remained similar up to age 11. The increasing trend of executive dysfunctions in children with psychosocial disabilities underscores the necessity for early intervention and support programs for these children.

Differences were observed in the patterns of executive dysfunction changes according to specific areas of psychosocial disabilities. This information could serve as foundational data for developing support systems tailored to children with these conditions. Children with psychosocial disabilities, especially, showed a tendency for increased behavioral control difficulties compared to their counterparts. Therefore, offering behavior regulation training programs to children experiencing psychosocial disorders could be beneficial.

This study utilized both Bayesian repeated measures analysis and repeated measures analysis of variance. Through these two methodologies, somewhat different results were obtained. For instance, while the repeated measures analysis of variance captured both interaction effects and main effects between psychosocial disability and changes in executive dysfunctions, the Bayesian repeated measures analysis did not provide sufficient evidence for interaction effects. This might suggest the need for utilizing alternative research methods for studies targeting minority groups. Although the children with psychosocial disorders of interest in this study constituted only 1.79% of the total case subjects, amounting to 22 individuals, more sophisticated statistical results were obtained using Bayesian repeated measures analysis. Bayesian analysis combines prior distribution with likelihood obtained from data to estimate posterior distribution, known to be more flexible and suitable for handling complex data structures than traditional approaches [28]. This signifies the value of providing a complementary methodology capable of yielding meaningful research outcomes for rare disorders or cases.

### Hypothesis generation

The conclusions drawn from this study should be regarded as hypothesis-generating rather than providing direct policy-making implications at this stage. While the analysis offers valuable insights into the developmental patterns of executive dysfunctions and their associations with psychosocial disabilities, further research is required to confirm these findings and develop specific policy recommendations. The ongoing research and future studies will be crucial in translating these preliminary insights into effective mental health policies and child welfare programs.
